# Realist evaluation of public engagement and involvement in data-intensive health research

**DOI:** 10.1186/s40900-020-00215-4

**Published:** 2020-06-29

**Authors:** Georgina Hobbs, Mary P. Tully

**Affiliations:** 1 Evidence and Research Manager, Manchester Health and Care Commissioning, Parkway 3, Princess Rd, Manchester, M14-7LU UK; 2 Division of Pharmacy and Optometry, School of Health Sciences, Faculty of Biology, Medicine and Health, University of Manchester, Manchester Academic Health Science Centre, Manchester, UK

**Keywords:** Impact, Patient and public involvement, Public involvement, Realistic evaluation, Personal development

## Abstract

**Background:**

High quality public engagement and involvement (PEI) in data-intensive health research is seen as one way of ensuring that social legitimacy, i.e. a social license, is conferred through public acceptance of the need for research use of their data. This is a complex research area, and portfolios of involvement have been suggested, but not yet evaluated, to support the role of public contributors. The study aim was to evaluate if and how membership of a data-intensive research public forum can act as a mechanism for enhancing members’ personal development. Our objective was to understand the circumstances and mechanisms that help to explain how, why and for whom involvement with a public forum enhanced those members’ personal development.

**Method:**

Qualitative data were collected from 15 current and previous members, via semi-structured interviews, notes from meetings, and consultations with and feedback from members. Data were critically compared, contrasted and reviewed until no new themes could be discerned and then condensed into context-mechanism-outcome (CMO) configurations. Realist evaluation was used to generate a theoretical and empirical appreciation of the contextual circumstances and mechanisms which help to explain the extent to which involvement with a public forum would enhance members’ personal development and, if so, how, why, and for whom.

**Results:**

Three CMO configurations were identified. All of them showed that using the portfolio facilitated growth in forum members’ personal development, but only where the members valued using the portfolio. This was particularly so for female members. Members valued the portfolio in one or more of three ways: as a tool to record and evidence activities, to facilitate reflective practice or as a guiding framework.

**Conclusions:**

Data analysis and consideration of the three CMO configurations suggests a refined middle range theory that ‘The use of a portfolio as a framework for learning in a public forum will facilitate members’ personal development if they value its use as a framework for learning’. Further work is needed to confirm these findings both elsewhere in data-intensive health research and in other complex research areas using public forums for PEI.

**Plain English summary:**

Public engagement and involvement in health research is now well established and makes a valuable contribution to the research process. However, little is known about its impact on participants. This article investigates how involvement in a data-intensive health research public forum impacts on public forum members, rather than the research process. Personal involvement portfolios were used to support their involvement work and help evaluate if and how involvement in research activities enhanced members’ personal development. Taking a realist evaluation approach, ‘Context-Mechanism-Outcome’ configurations were used to explore how membership of a public forum might enhance public forum members’ personal development. The Context-Mechanism-Outcome configuration refers to an exploration of what influences the extent to which an intervention is successful or unsuccessful in producing positive outcomes and tries to identify the reasons why it is successful for some and unsuccessful for others. However, evidence from this realist evaluation recommends that engagement and involvement should always be underpinned by procedures which ensure that public contributors receive ongoing and tailored guidance and support throughout the process.

## Background

Research being carried out ‘with’ or ‘by’ members of the public (rather than ‘to’, ‘about’ or ‘for’ them) is playing an increasingly important part in improving the conduct and quality of research [1–4]. For example, it can improve participant information materials and increase participant recruitment and retention [1, 5]. Such collaborative input by public ‘contributors’ has various terms, depending on the context – for example, internationally it is called public involvement in the United Kingdom (UK) [6], public engagement in Canada [7] and public participation in the United States of America [8]. Even within the UK, the term ‘public engagement’ alludes to different purposes and activities to researchers coming from a different discipline, such as data science, social science or health research [6, 9]. We describe any type of such general collaborative input in this paper as “public input”. Within the multidisciplinary data-intensive health research (i.e. “research conducted through linkage and analysis of data from one or more sources, especially health-related data”), the term used internationally to describe this input is public engagement and involvement (PEI) [10, 11]. By this term, we mean a variety of activities for the purposes of “raising awareness of current research, consulting members of the public on their views about health research, working in partnership, to empowering members of the public to play a role in shaping current or future research or governance practices” [11].

Data-intensive health research may not appear to need public input at first glance. After all, data scientists typically have no contact with their large numbers of “research participants” whose data they analyse; they do not need participant information sheets, nor to ensure recruitment or retention of those participants. However, high quality PEI in data-intensive health research is seen as one way of helping researchers deliver information and engage in public dialogue to ensure a social license from the public that accepts the need for the research use of their data [12]. Recently, there has been international consensus that PEI is “a key part of the solution to establish socially beneficial data-intensive health research for all” [10].

The impact of public input in research generally has tended to be evaluated using assessments of the impact on the research itself [1] or on researchers [5, 13]. A large realist evaluation has also investigated the key contextual factors and mechanisms that lead to the desired impact on research [14]. However, acting as a public contributor can also have personal benefits for these individuals. A recent systematic review of other systematic reviews of public input into clinical trials has highlighted these ‘internal benefits’. For example, the patients acting as public contributors may feel listened to and empowered, and gain knowledge about their condition [15].

High quality and meaningful input is often delivered using public groups or forums, both in clinical [3, 5] and in data-intensive health research [16, 17]. Such public forums act as an interface between the public and researchers, in order to enhance research quality and impact [15, 18]. Given there is evidence that involvement in other types of forums can facilitate growth of members’ personal development [19, 20], it is probable that research-focused public forums also offers personal benefit to members.

However, membership of a PEI forum for data intensive health research initiative may not be, on its own, sufficient to ensure personal development. Using a portfolio, such as the Involvement Portfolio developed by the National Health Service (NHS) Research and Development Forum User and Carer Working Group [21] may further facilitate personal benefit to members. The NHS portfolio enables public contributors to create a record of the skills and expertise they have gained from their work [22] and is recommended for public contributors by the English national public involvement organisation INVOLVE [23]. The NHS portfolio has not been formally evaluated. However, using such a portfolio may enable members to deliver high quality input through an enhanced sense of power, control and active influence in the process brought about because the use of portfolio is predicated on the premise that the owner has the capacity and autonomy to communicate, reveal and disclose their personal evidence at their discretion. This has been widely reported in research which uses diary methodologies [24, 25].

PEI in data-intensive health research usually recruits members of the public [16, 17, 26], rather than the patients or carers who typically provide public input in clinical trials [15]. In addition, PEI in data-intensive health research is seldom described in the literature [16, 17, 26] and has rarely been formally evaluated [16]. Little is known about how membership of public forum affects aspects of personal development nor about the causative underlying mechanisms that might facilitate enhanced personal development through this membership or using a portfolio. Thus, more needs to done to help understand and contextualise the impact that membership of a public forum has on the members themselves.

### Aims and objectives

The aim of this study was to evaluate whether membership of a data-intensive research public forum can function as a mechanism for enhancing members’ personal development. The public forum was part of a broader Connected Health Cities project, which is described in more detail below. Throughout the article, those members of the public who joined the forum will be referred to as ‘public forum members’ or simply ‘members’. Realist evaluation was used to generate a theoretical and empirical appreciation of the contextual circumstances and mechanisms which help to explain the extent to which involvement with a public forum would enhance members’ personal development and, if so, how, why, and for whom.

## Methods

### Research design

Quantitative evaluations that evaluate the impact of PEI have predetermined endpoints, and thus lack the ability to evaluate unexpected outcomes (as the researchers ‘don’t know what they don’t know’) and do not take into account the context within which the evaluation data were generated [5, 27]. A more appropriate method of evaluating such outcomes is realist evaluation. This approach aims, broadly, to conduct an evaluation with a view to understanding what works, for whom, and under what circumstances [28–30]. Thus, it moves beyond traditional evaluation of the efficacy of the process or intervention being evaluated and is also concerned with providing a structure that facilitates the ongoing testing and refinement of the evaluation theory associated with the intervention.

Realist evaluation generates what is known as a middle range theory (MRT), described as an “account of the processes that explain how an intervention leads to a particular outcome” [29]. These MRTs can be constructed either from hypotheses that are tested and refined [29, 30] or (as we have done) from preliminary MRTs developed from the literature, which are tested as part of a realist evaluation and end with a more refined MRT [28]. Such iterative cycles of inquiry and refinement enable the development of a stronger theoretical appreciation of the contextual conditioning which underpins the success or failure of the intervention or programme being evaluated. Our preliminary MRT, based on the literature outlined above, proposed that a public forum which used a portfolio as a framework for learning would enhance members’ personal development.

Realist evaluation involves determining how aspects of a project have performed within its specific milieu and environment. The choice of data collection, analysis methods and tools for this evaluation was guided by the types of data needed to answer the evaluation questions.

### Setting

The setting for the evaluation was the Connected Health Cities project. Connected Health Cities was a Department of Health funded pilot programme [31], which aimed to transform healthcare across four regions in the North of England by using healthcare data to improve the delivery and outcomes of selected care pathways. The Connected Health Cities’ PEI strategy focused on gaining public trust that their researchers were using health data responsibly, safely and to improve services for the benefit of all patients. The objective of the strategy was to seek public engagement and involvement in Greater Manchester Connected Health Cities’ use of transformative healthcare solutions. A public forum was chosen as one of the main mechanisms for PEI in the Greater Manchester region. Concomitantly, the forum also offered public forum members the opportunity to work alongside Greater Manchester Connected Health Cities researchers to debate the merit of both initial research ideas and more developed research proposals, as described below.

### Public forum and public forum members

The forum was created in 2017 and ran for 18 months, until Connected Health Cities started to wind down. Public forum members were recruited via widespread advertisement and final selection of 12 members was based, in part, on their commitment to being actively involved in the evaluation process. Three members left the group in the first year and were replaced by three reserve members. Throughout, there were five women and seven men in the forum, three of whom were under 25 years of age. The profile of membership was ‘students’ (two), ‘retired’ (initially seven, one of whom left and was replaced with someone employed) and ‘employed’ (initially three, and then four). Data from all 15 public forum members were included in the analysis.

The members were provided with materials designed to facilitate their learning, personal development, and ability to contribute fully to Greater Manchester Connected Health Cities research, public engagement activities and the evaluation process. These materials related to both data-intensive health research, research methods and approaches underpinning evaluation processes and personal development. Members were also provided with individualised support for the evaluation process, such as personal one-to-one contact, electronic and telephone consultations, and training opportunities, for example Massive Open Online Courses.

The members were encouraged to keep a personal involvement portfolio, developed by the NHS Research and Development Forum User and Carer Working Group [21], but its use was not mandated. The portfolio was designed to provide participants with a framework that enabled them to: identify their developmental needs and provide feedback by recording information about what they thought of the PEI process (especially what worked, did not work and could be improved, and whether the process was balanced and inclusive) and how it could be improved (Table 1). The portfolios were amended and updated to document members’ personal development, increased awareness and empowerment and attainment of transferable skills and knowledge acquisition. The use of portfolios is associated with the development of reflection [25, 32]; however, it is widely acknowledged that developing one’s reflective practice is challenging [33]. Public forum members were, therefore, supported individually to practice their reflective skills, and took part in an interactive presentation which showcased examples of how to develop reflective practice.

**Table 1:** Components of personal involvement portfolio used by public forum members [21]

Component	Details
Personal Profile	Personal details including education, qualifications and employment
Relevant Experience	Volunteering and personal experience
Training Record	Training events attended and events where been trainer or facilitator
Personal statement	Overall description of skills and experience they may have gained from involvement activities
Involvement activities	Summary of each activity, skills and experience gained, evidence such as certificates or feedback and personal reflections on their involvement in this activity
References	Details of relevant individuals and how known to the public contributor

There were seven meetings of the forum over the 18 months. Overall, the PEI provided by the forum can be classified as mainly ‘consult’ and ‘involve’ and occasionally ‘collaborate’ on the international Spectrum of Public Participation [8]. Members contributed to a number of Greater Manchester Connected Health Cities research activities, and to other aspects of the project that were of particular relevance to their individual interests. Examples included iterative involvement in the design of a survey to determine public acceptability of patient focused interventions to reduce antibiotic prescribing, commenting on a proposed research collaboration with a commercial company concerned with interventions for stroke patients and attending science festivals with the PEI staff. Members’ evaluation of how these activities and events contributed to the aims and objective of the Connected Health Cities PEI strategy were captured in their portfolios through reflective writing. This paper, however, focuses on the evaluation of public forum membership and its impact on the personal development of the members.

### Data collection

Most data were qualitative and collected by the first author (GH) over the life course of the forum. Soon after recruitment, members were asked to reflect upon and document what they hoped to learn and take away from their involvement in the forum. This provided a baseline assessment, which was used by GH and each individual member to help understand and plot their ‘journey’ through the life course of the public forum. Data were collected formally via semi-structured interviews with 10 existing members, exit interviews with three members who left the forum and field notes from the forum meetings and informally via emails, personal communications and feedback from public forum members. The forum meetings and semi-structured and exit interviews lasted between 30 and 90 min, depending upon each individual public forum member. Field notes were taken during forum meetings, which allowed GH to record, for example, how members interacted with each other, the topics under discussion and manifested signs of self-confidence, self-awareness, critical thinking and communication skills. Interviews were not recorded but were documented in detail and the notes distilled and condensed into a summarised account of the session.

Public forum members also contributed to data collection via personal one-to-one consultation sessions and a final summative public forum meeting at the end of the project. During the consultations, the researcher and the individual member worked together to draw out understandings of the outcomes and impact of the project. Collectively, the consultations and the final public forum meeting drew upon the members’ data and the learning which they had built up over the course of the project to assess the overall value, successes and failures of the project.

The portfolio played an important dual role of providing the members with a tool to record their involvement and to note reflections, and providing ongoing data to appraise how best to support their involvement. Members contributed selected reflections from their portfolios, which were submitted for group discussion to the public forum meetings.

### Data analysis

Field notes were transcribed, read for general comprehension and combined with the data from the questionnaires, interviews and emails, then read thoroughly. The data analysis was primarily undertaken by GH who used inductive and deductive coding to structure the process [34]. The first stage used deductive coding, where the data were examined to establish whether themes or theories of practice that had been observed in previous research or theoretical accounts were apparent here. The second stage used inductive coding, i.e. the data were examined to investigate areas of commonality and divergence and to ascertain if there were patterns or relationships between different elements of the data. The coding process was conducted iteratively, consistent with qualitative realist evaluation [28, 35].

Following these two stages, the data were examined using thematic analysis to identify, clarify and coalesce the data into provisional themes. These themes were generated based on the identification and coding of recurring words, expressions, topics, concepts and subject matter associated with personal development. Field data were used for triangulation and to add a layer of nuanced understanding to the provisional themes generated from analysis of the other data.

The preliminary analysis and provisional themes were shared with the members during forum meetings, and the members assessed and discussed the analysis and interpretation of the data iteratively throughout the life course of the project, enabling data immersion and crystallisation, i.e. a process that distils understanding from text, until no new themes could be identified in the data. Consistent with qualitative realist evaluation [36] and analytic generalisation [37], those themes sharing common characteristics were combined., refining them three broad thematic areas - the roles of the portfolio as a recording tool, as a reflective tool and as a guiding framework. Using Pawson and Tilley’s approach to data analysis [28], the themes were then investigated using data exploration, analysis, cross-referencing and reflective practice in order to conceptualise and construct the data into linked context-mechanism-outcome (CMO) configurations (C + M = O) which are widely used as the main structure for realist evaluation [28–30]. The three resulting CMO configurations, whilst broadly distinct, contained some overlapping characteristics, a result that is not untypical in the evaluation of real world settings [28] and reflects the often complex relations between different components of an intervention [38]. In keeping with the theoretical principles of realist evaluation, the resulting configurations were then used to refine the original MRT.

### Trustworthiness

To ensure trustworthiness of the data analysis, several approaches were taken [39]. As described above, all data were signed off by the specific individual when it related to their own activities and data from forum meetings were signed off by the group collectively. The public forum members contributed to the analysis iteratively as data were collected. These ensured credibility of the results from the perspective of the members themselves. Detailed descriptions of both the context and the data analysis process are given, to allow for transferability and dependability.

## Results

Overall, data were collected from all the 12 inaugural members and the 3 replacement members, although a complete dataset was not available for some. There were missing responses from two members and a further two members did not complete the introductory questionnaires; three of these four members were men.

Three CMO configurations were identified from the data, as shown in Table 2. All showed that using the portfolio facilitated growth in members’ personal development, but only where the members valued using the portfolio. The three CMO configurations are numbered below in the order by which they were identified; the numbering, however, does not denote ranked importance.

**Table 2:** CMO configurations from the realist evaluation

Configuration	Title	Details
1	Valuing the portfolio as a tool to record and evidence activities	When members value the portfolio as a record of achievement **(C),** personal motivation to record and map transferable skills **(M)**, facilitated enhanced skillset, especially in newly acquired skills **(O)**
2	Valuing the portfolio as a tool to facilitate reflective practice	When female members had previous experience of using reflective practice in a professional capacity **(C),** the personal motivation to adopt reflective practice to supports ones’ personal development **(M)** led to enhanced self-confidence and self-awareness **(O)**
3	Valuing portfolio as a guiding framework	When participants had a positive perception of the portfolio as a guiding framework **(C)** there was a willingness to use the portfolio **(C)**. This ultimately enabled reflective practice **(M)** which enhanced reflective experiential practice, critical thinking and improved communication skills**(O)**

### Configuration 1 – valuing the portfolio as a tool to record and evidence activities

This configuration suggests that, when members valued the portfolio as a record of achievement **(C),** personal motivation to record and map transferable skills **(M)**, it facilitated an enhanced skillset, especially in newly acquired skills **(O).** The enabling mechanism was a positive perception that their portfolio would be useful in helping them obtain additional public service roles and or employment. Many of those in the public forum were keen to use their involvement as a stepping stone to membership of other public involvement groups. Thus the portfolio was useful as a document that summarised members’ progression throughout their involvement in the public forum. It was seen as providing members with a record and proof of what they had learnt and the skills they had developed. It was valued for use in applications and at interviews as means of demonstrating their employability and or a broader commitment to career development or societal advancement. For some members, primarily men, the portfolio acted as a living document which showcased how involvement in a public forum had made an important contribution to gaining practical skills that they could use in real world settings.

*“If I were to apply for a role on another Health related forum or committee for example, the PIP [personal involvement portfolio] will show: - a desire to improve my knowledge and willingness to learn and develop”. (Member A, Male, interview)*

Two individual level contextual factors were identified as facilitating this mechanism. The first was a perception that the portfolio could act as a framework for continuing professional development i.e. that it could be employed as a tool in which the attainment of personal skills could be mapped against the requirement for external professional standards.

*The portfolio will be really useful for my career development because it’s a record, of what I have done in my own time to improve my skills. This will enhance my employability because it demonstrates my commitment to learning new skills and ongoing development, things that are important in a professional development capacity. (Member C, Male, email)*

The second was that the portfolio helped members identify gaps in their knowledge and this awareness encouraged some members to undertake additional study to improve their learning.

*The PIP [personal involvement portfolio] has been important because I’m keeping a record of all my learning and development that’s specific to GM CHC [Greater Manchester Connected Health Cities] and it has helped me spot where I'm a bit lacking in knowledge and so I have tried to address that so for example I have completed a MOOC [Massive Open Online Course] about Antibiotic Resistance and know so much more now. (Member B, Male, interview*

This subsequent additional learning frequently enhanced the members’ self-confidence and inspired greater involvement in public engagement events and activities within the Greater Manchester Connected Health Cities project.

However, not all the members valued the portfolio as a tool to record and evidence activities. The face to face consultations and exit interviews identified that the oldest members of the forum were less enthusiastic about the portfolio than the other members. These members were all retired and this may have influenced their attitude towards this aspect of the portfolio as they may have felt they had little reason to catalogue their activities, within the broader context of this being useful in obtaining employment or additional public service roles.

Exit interviews with the three members who left the forum early provided a rich data source in which to explore how initial involvement in a public forum influenced members’ personal development. For these members, the portfolio played only a small role in enhancing their personal development but these skills had contributed to an improved sense of how to work effectively in a team or sharpened their reflective aptitude.

*“I don’t think I really had the time to pick up new skills but writing down my personal statement did help me to appreciate that I had become a better listener and also that using the PIP [personal involvement portfolio] was helping me hone my reflective skills”. (Early exit member1, Female, exit interview*

Many members (*N* = 7, five of whom were women), however, thought the portfolio was very useful for documenting their self-development. The portfolio worked well because it facilitated the development of personal skills, provided members with a hard-copy record of involvement and was an effective way of learning and developing both individually and for the programme. However this appeared to be linked to gender and previous experience of using reflection in a professional capacity. The men primarily valued the portfolio for its utility as a record of achievement.

*“The PIP [personal involvement portfolio] is a useful document for anyone wishing to apply for work or any voluntary role. It is a good document to support any application as it shows commitment, willingness to learn and contribute as well to self-development”. (Member D, Male, interview)*

Female members, however, also valued the portfolio because it facilitated personal learning and development, particularly those who had previous experience of using reflection in a professional capacity.

*I have experience of evaluating my learning in a number of ways. For example in the courses I have undertaken in [name of company], maintaining a reflective journal was an essential learning tool and contributory element. Self-awareness has been engendered into both my formal learning and operational work. So writing it down is not new to me. I am also an organised person and like to review what I have done, reflect, so I can identify learning points to fulfil my drive to develop myself. (Member E, Female, interview)*

The degree to which gender may affect members’ relationship to diary based approaches to learning and development will be considered in the discussion.

The single most important outcome reported by eight members was increased self-confidence in their ability to enhance existing skills (particularly their communication skills) and acquire new skills. This was typically described as a growing sense of belief that they could take part and be effective in public engagement activities. During the first year of the forum, members did not feel confident enough to help out at public engagement events. In the final 6 months of the project, their developing self-confidence saw some members participate in public engagement events, engaging in discussions with the public, running activities with school children and co-designing and delivering workshops for young people.

*Even though I am used to leading, facilitating and generating discussion amongst groups and individuals when we started I didn’t feel confident about going out and getting involved in the public engagement activities. I've grown in confidence loads though and I have done loads of different activities and events this year and loved doing them. (Member J, Female, portfolio)*

Data from the face to face consultation sessions revealed that female members were particularly effusive about the affect the portfolio had on their transferable skills and the broader application of the newly acquired skills to other aspects of their lives. For some this meant taking part in digital activities which they had not previously come into contact with or did not have the confidence or expertise to tackle. For others it also encouraged health and wellbeing related behaviour change.

*“It has encouraged me to apply knowledge acquirement to things I could not do before – taking part in webinars, using doodle poll e.g. joining the on-line [discussion] group for the [public engagement activity] was very self-motivating and helped me improve my fitness”. (Member F, Female, portfolio)*

Overall, it may be that this very pragmatic aspect of a member’s involvement in a public forum can help to demonstrate how the acquisition of tangible skills contributes to personal development.

### Configuration 2 – valuing the portfolio as a tool to facilitate reflective practice

This configuration suggests that, when female members had previous experience of using reflective practice in a professional capacity **(C),** the personal motivation to adopt reflective practice to supports ones’ personal development **(M)** led to enhanced self-confidence and self-awareness **(O)**. The data suggested that, whilst many members used reflection in some capacity, e.g. to demonstrate lessons learnt, only certain members used the portfolio as a mechanism that enabled and structured reflection and reflective learning. All three younger members were either studying, had recently completed their studies or were going back to studying; reflection formed or had formed part of their learning programme. However, it was notable that the two young men used reflection at a superficial level, primarily to help them identify skills gaps, as part of their broader commitment to enhancing their employability. The younger woman, however, engaged in critical reflection as a means of supporting and facilitating her ability to engage in challenging PEI pursuits and gain meaningful insights.

The data suggested that it was only female members, especially those who had previously used reflective practice (as quoted above), who actively valued reflection and pursued it to the point when they became capable of critical reflection. When they had attained this level of reflection, they described how this process had enhanced their personal development.

*I was used to being reflective in my professional life but I am retired so it had been a while since I had used these skills. I always knew that it was just a case of working to reignite my skills. I have done that and acquired new skills as well. (Early exit member 2, Female, exit interview)*

Analysis indicated that acquiring active listening skills was an important personal outcome for members for a number of reasons. It helped them build and maintain relationships with fellow public forum members, had a positive effect on their behaviour in forum meetings, workshops and public engagement activities and allowed them to see how much they could learn from others if they actively listened to and focused on the speakers’ message and point of view. This inspired members to be more considered in their response to others, a skill used to good effect when discussing use of health data with members of the public at public engagement events.

Analysis indicated that those who attained an enhanced level of self-awareness concurrently became more aware of their working relation with others. For example, collaborative working skills was an equally important outcome i.e. having learnt to employ their active listening skills, members communicated their point of view more effectively, accepted and promoted compromise, made collective decisions, became more open-minded, and successfully built and maintained functioning relationships with others. On a personal level, members felt that they also had a greater appreciation of the importance of embracing diversity of opinion, being respectful of views inconsistent with their own and valuing the skills, experiences, and contributions of others. The members reported that enhanced self-confidence and self-awareness, combined with the newly acquired or improved communication skills, could be used to good effect in other public service roles.

*The PIP [personal involvement portfolio] really helped me get to grips with reflecting on my own behaviour, especially in meetings. I realised that I never truly listened I was just waiting for my opportunity to speak but that meant I wasn’t learning from others. That penny dropping was important because when I actually started to listen I realised how much I could learn from the other members of the forum. (Member E, Female, interview)*

### Configuration 3 – valuing the portfolio as a guiding framework

When members had a positive perception of the portfolio as a guiding framework **(C)** there was a willingness to use the portfolio **(C)**. This ultimately enabled reflective practice **(M)** which enhanced reflective experiential practice, critical thinking and improved communication skills **(O)**. The enabling mechanism was that personal motivation to adopt reflective practice in order to support personal development

*So the forum has provided me with the medium to practice my skills, particularly circumspection. I haven’t always got it right but reflection allows me to recognise that so I can make adjustments. (Replacement Member 1, Female, portfolio)*

One, individual level, contextual factor identified as facilitating this mechanism was a perception that reflective practice could be harnessed to improve or attain certain skills which could feedback into society. The desire to make a positive contribution to research, public health or society motivated many to work through the challenges of developing critical reflection skills.

*I want to give something back and getting involved in this project is a great way to do that. I also think it’s very important for the voice of the public to be included … and so although it has been time consuming and very hard at times I'm glad that I didn’t give up on the portfolio and my attempts to be more adept at reflection! (Member H, Male, email)*

Ten members (seven of whom were women) thought that the portfolio had been helpful in encouraging such reflection. Face to face consultation sessions and exit interviews facilitated our understanding that being more reflective was part and parcel of how most members began to appreciate that the diary approach underpinning the portfolio enabled their personal development. By becoming more reflective, members became more aware of aspects of their personal development that they wished to enhance and made a strong commitment to learning in order to progress as an individual and as part of the team. However, it was notable that for others, primarily the men, the portfolio was used predominantly as a diary as a record of achievement.

*For me personally I've not put that much effort in or looked at it outside of the meetings (Member I, Male, email)*

Internalised motivation appeared to underpin the key enabling mechanisms. Five members’ data identified motivation for personal development achieved through reflection as the primary catalyst for continued use of their portfolio. For all these member, motivated use of their portfolio related to using their new skills and learning for the benefit of the project and the broader public good. For those who were retired, the motivated use also related to building on their former professional practice in order to continue to develop as a person outside of the workplace. For those who were not retired, it also related to their personal and professional development.

Whilst these individual level contextual factors played an important role as enabling mechanisms, societal level contextual factors also played a part. The female members appeared to find reflection easier than their male counterparts. This was underpinned in part by previous experience of using reflective practice in a professional capacity, as described earlier. Analysis of all the strands of data revealed that female members were more motivated to use the portfolio and this was enabled by prior use of reflection in a professional capacity, which meant that they valued reflective practice. This prior use accelerated their competence in undertaking critical reflection and the subsequent enhancement of their personal development.

Members who used the portfolio as a means of guiding their learning and development demonstrated increased self-confidence and critical thinking, notably in their ability to undertake tasks that were previously unfamiliar to them. In particular, these were activities associated with digital technology (*N* = 5) and where members felt more able to assert themselves in public and professional arenas (*N* = 4). It was in these aligned domains of personal development that members recorded the greatest impact. One member cared for her mother and was responsible for assisting with her interactions with health professionals. Previously, meetings with her mother’s consultants had left the member feeling powerless and her voice ignored. During her semi-structured interview, this member noted that her enhanced self-confidence, ability to apply critical thinking (so as to impress upon the clinicians the value and contribution that her lived experience brought to the consultation), improved communication skills, greater knowledge and understanding of healthcare and the sense of authority to assert herself, enabled her to challenge her mother’s consultant about her treatment. As a result, valuable new information was discussed with the consultant, who listened to and acted on the information. This led to an improved health outcome for her mother.

*I definitely feel able to challenge health professionals now. In fact I have done so on a number of occasions now and they have listened to me! (Member G, Female, portfolio)*

Three women identified the portfolio as the most successful element of their involvement in the public forum as it had enabled them to develop personal and professional insights and encouraged additional and improved learning behaviours. This may have been achieved because the very nature of writing and critically reflecting on one’s thoughts in a journal facilitates the latter’s ability to understand how theory and practice inform each other, in effect acting as bridging concept or connection between theory and practice.

*“Playing devil’s advocate to my own theories, considering how my behaviours impacted on others. I could do this is an oral way but that only captures the moment and I need to be able to recall and revisit learning. Learning evolves and therefore needs to be reviewed. Actions implemented, theories tested, amended adopted or rejected and changes made. I can only make my learning meaningful and capable of meeting these objectives if I keep a written record”. (Member G, Female, portfolio)*

Nearly half of the members (*N* = 6, including four women) also reported that the portfolio was a useful mechanism for cascading elements of what they learnt to other public forum members and members of the broader public.

*The portfolio encouraged me do a few MOOCs [Massive Open Online Courses] and I was able to take what I had learnt from those and share it with the rest of the group and my family and friends (Member B , Male, portfolio)*

However, six members thought that the portfolio was ‘*not at all useful*’ to share with others their learning, skills or knowledge gained. This was reported regardless of demographic characteristics such as gender, age and employment status.

## Discussion

This small realist evaluation found that, in general, membership of a public forum for a data-intensive research initiative has enhanced some of the members’ personal development. The mechanism for this was the use of a personal development portfolio, which was valued by the members as a tool to record and evidence their PEI activities, to facilitate reflective practice and as a guiding framework for personal development. These findings were particularly noticeable for the female members. In keeping with the realist evaluation methodology, consideration of the three CMO configurations that were identified suggests, therefore, that the MRT should be refined as ‘the use of a portfolio as a framework for learning in a public forum will facilitate members’ personal development if they value its use as a framework for learning’.

There is some evidence that such portfolios successfully prompt insights, encourage further study and improve learning because they act as a bridging concept between theory and practice, thereby aiding understanding of how theory and practice inform each other [40]. Others suggest, however, that the diary method, which is predicated on a very personal and private recollection of one’s inner most thoughts, may not conducive to the more collective communication of learning to others [32, 40]. The portfolio provided members with the motivation to use reflection as a means to embed learning into their daily lives, as well as in the applied work of the public forum. Those who embraced the portfolio and used it on a regular basis reported that they had become more self-assertive and felt a greater sense of agency, particularly in relation to their health and that of their significant others. The use of portfolios in public forums is under researched; however the fact that the portfolios encouraged forum members to undertake additional study to improve their learning may be explained by similar findings reported in other sectors. For example, Beecher and colleagues reported that the use of an education based portfolio stimulated reflective practice amongst medical professionals [41]. Similarly, a literature review undertaken by Mann and colleagues reported that health professionals who chose to use portfolios were already positively inclined to reflection and perceived its usefulness and value in helping them reach particular goals [42].

There was a strong gender difference between those who valued the portfolio as a tool to structure reflective practice and to share learning and those who did not. Our findings suggested that women were more likely to use the portfolio because they valued reflective practice and men were most likely to use it because it provided them with a record of involvement which could be used for employment purposes and was an effective way of learning and developing both individually and for the project. This may reflect the well-established evidence base of gendered differences in preferred learning styles and strategies [43, 44]. However, it may also reflect the equally well-established evidence base that there is a gender and socio-cultural bias within the delivery of public input, where it has been shown that women, particularly in higher socioeconomic groups, were more willing to volunteer as public contributors [45]. The issue of representation in public input is frequently made more challenging when considered alongside issues such as remuneration, language, or access, all of which can act as barriers to those from poorer backgrounds or different socio-cultural backgrounds to become public contributors [46, 47].

However, the degree to which gender affected the members’ relationship to the use of the portfolio is open to conjecture, given the size of this study, and further research is needed to investigate this. It may be related to the working environment into which the public forum was embedded. The PEI researcher who lead the group (GH) and the other institutional PEI leads (including MPT) were all women. Research which explores gendered differences in the workplace suggests there is evidence that some men find it hard to admit they need development and find it difficult to self-analyse and identify weakness or areas where they could develop [48, 49]. These challenges may have been amplified by a disinclination to admit this to the female public engagement leads in this study. Concurrently, this gender biased environment may have produced social relationships which were more supportive of the use of reflection and reflective practices amongst the female members. Previous research also suggests that this finding may be due in part to societal processes that mean that reflective practice is more commonly associated with professions predominantly staffed by women and reflection is more commonly associated with psycho-social behaviour assigned to women [32, 48, 50, 51]. Nursing, for example, has mobilised reflective practice is a key aspect of on-the-job learning and development [52].

Although there have been other realist evaluations of PEI [14], this is one of the first to focus on the personal development of members of a public forum. The findings offer an appreciation of how public forums can be mobilised to enhance members’ personal development. Realist evaluation is primarily concerned with clarifying causality within localised contexts [53]. Therefore, our findings do not purport to be representative or generalisable to other settings at this stage, but generate a more refined MRT that could be tested in future studies.

This study is important because an increasing emphasis has been placed on PEI in research [4]. PEI offers people the opportunity to, among other benefits, develop skills, broaden their horizons and (in some instances) earn money [54–56] and so these positions are highly sought after [57]. However, there are concerns that lay people recruited to PEI roles have ‘worked’ in this field for a number of years and are so ingrained in the system that they no longer offer an authentic ‘lay’ perspective [57, 58]. Ives and colleagues refer to this as the ‘professionalisation paradox’ [57]. The field of PEI therefore needs to expand who and how it recruits to PEI roles. A systematic review of structures designed to support PEI in research [59] grouped them into five categories: power focused, priority setting, study focused, report focused and partnership focused. None of the structures could be categorised as having a personal development or social impact focus for the individuals who contribute to PEI in research or the broader public. However, it has been argued that personal development in PEI should be seen as incidental to the impact of PEI on the research or researchers [57], which may explain this gap.

The refined MRT after our data analysis offers an example of a theory that focuses on personal development and social impact. It also offers up a model in which the ‘professionalisation paradox’ can be mitigated by broadening the appeal of PEI membership to people in society who could benefit the most from involvement in public endeavours but may not traditionally think of ‘working’ in PEI. This might ensure that forum members are drawn from a broader sector of the population and thus deliver an authentic lay perspective [57]. Our findings suggest that, if organisations involved in PEI want to recruit people with a fresh perspective and or focus on delivering around social value/impact, they should prioritise the personal benefits that involvement in PEI can bring to participants and the broader benefits that can accrue to society. These may be benefits that, for example, relate to gaining practical skills that could improve employability outside of PEI or facilitate community led regeneration initiatives. In addition, regardless of which approach is adopted to underpin PEI in research, evidence from this evaluation concurs with other studies which conclude that public input should endeavour to be underpinned by procedures that ensure that those involved in the process (be they researchers, clinicians or members of the public) receive ongoing and tailored guidance and support throughout the process [57–59].

A key strength of the methodological approach was the involvement of the members themselves in the initial stages of the data analysis, which ensured the credibility of the findings. Data were collected from a range of sources (e.g. interviews, portfolios and field notes) which allowed triangulation. However, it is important to note that the members’ level of engagement and involvement was circumscribed not only by the parameters set by the project but also by their perception of and confidence in their individual skill-set, knowledge and expertise. No public forum members were involved in the writing of this article, as those invited found the realist evaluation process complex and beyond their skill-set and they declined to take part. Unfortunately there were missing data from four of the members, which potentially could have impacted on our findings. In addition, some members did not use the portfolio consistently. During interviews for public forum membership, the importance of maintaining the portfolio was communicated to potential members and those who seemed amenable to this were prioritised for recruitment. A prescriptive approach, which used ongoing direct instruction to scrupulously use and maintain the portfolio, was purposefully not adopted. Ascertaining whether the portfolio would support personal development required that it be adopted and maintained willingly and without pressure.

## Conclusions

This realist evaluation has shown that the use of a portfolio as a framework for learning in a public forum will facilitate members’ personal development if they value its use as a framework for learning. Therefore, if an organisation wants to enhance public understanding about complex research areas such as data-intensive health research, they could potentially underpin their PEI approach with a personal involvement portfolio and combine this with a realist evaluation of its impact. This will ensure that the modified MRT identified in this study can be fully evaluated and modified further if necessary.

## Supplementary information

**Additional file 1.** GRIPP2 checklist.

## Data Availability

Data cannot be shared as the members of the forum did not consent to this. In addition, the small number of members means there is a strong possibility that there would be content in the data that could identify individuals.

## Abbreviations

C: Context
CMO: Context-mechanism-outcome configurations
M: Mechanism
MRT: Middle range theory
O: Outcome
PEI: Public engagement and involvement
